# Rotational symmetry of the structured Chip/LDB-SSDP core module of the Wnt enhanceosome

**DOI:** 10.1073/pnas.1912705116

**Published:** 2019-09-30

**Authors:** Miha Renko, Marc Fiedler, Trevor J. Rutherford, Jonas V. Schaefer, Andreas Plückthun, Mariann Bienz

**Affiliations:** ^a^Medical Research Council Laboratory of Molecular Biology, CB2 0QH Cambridge, United Kingdom;; ^b^Department of Biochemistry, University of Zurich, 8057 Zurich, Switzerland

**Keywords:** Wnt enhanceosome, Chip/LDB1-SSDP, Pygo

## Abstract

The Chip/LIM-domain binding protein (LDB)–single-stranded DNA-binding protein (SSDP) (ChiLS) complex controls numerous cell-fate decisions in animal cells, by mediating transcription of developmental control genes via remote enhancers. ChiLS is recruited to these enhancers by lineage-specific LIM-domain proteins that bind to its Chip/LDB subunit. ChiLS recently emerged as the core module of the Wnt enhanceosome, a multiprotein complex that primes developmental control genes for timely Wnt responses. ChiLS binds to NPFxD motifs within Pygopus (Pygo) and the Osa/ARID1A subunit of the BAF chromatin remodeling complex, which could synergize with LIM proteins in tethering ChiLS to enhancers. Chip/LDB and SSDP both contain N-terminal dimerization domains that constitute the bulk of their structured cores. Here, we report the crystal structures of these dimerization domains, in part aided by DARPin chaperones. We conducted systematic surface scanning by structure-designed mutations, followed by in vitro and in vivo binding assays, to determine conserved surface residues required for binding between Chip/LDB, SSDP, and Pygo-NPFxD. Based on this, and on the 4:2 (SSDP-Chip/LDB) stoichiometry of ChiLS, we derive a highly constrained structural model for this complex, which adopts a rotationally symmetrical SSDP_2_-LDB_2_-SSDP_2_ architecture. Integrity of ChiLS is essential for Pygo binding, and our mutational analysis places the NPFxD pockets on either side of the Chip/LDB dimer, each flanked by an SSDP dimer. The symmetry and multivalency of ChiLS underpin its function as an enhancer module integrating Wnt signals with lineage-specific factors to operate context-dependent transcriptional switches that are pivotal for normal development and cancer.

## Significance

A structural model is presented for the core Chip/LIM-domain binding protein (LDB)–single-stranded DNA-binding protein (SSDP) (ChiLS) complex, which mediates long-range enhancer–promoter interactions to control the transcription of master regulatory genes with key functions in embryonic development, stem cell maintenance, and differentiation along adult cell lineages, including erythroid maturation. Because of its rotational symmetry and multivalency, ChiLS can bind to multiple combinations of lineage-specific DNA-binding proteins, as well as cofactors responding to extracellular signals such as Wnt, and is thus uniquely poised to integrate these inputs and translate them into transcriptional ON and OFF states of target genes.

Vertebrate LIM-domain binding protein 1 (LDB1, also known as NLI or CLIM) and its *Drosophila* ortholog Chip have pleiotropic functions in controlling embryonic and larval development at multiple stages (1–4), maintenance of intestinal and hematopoietic stem cells (5, 6), and differentiation along erythroid cell lineages (7, 8). Chip/LDB proteins are recruited to remote transcriptional enhancers of pivotal developmental control genes by their C-terminal LIM-interacting domain (LID) that binds directly to LIM-homeodomain DNA-binding proteins, or to GATA and basic-loop-helix (bHLH) DNA-binding proteins via LIM-only adaptors. They facilitate communication between remote enhancers and proximal promoters (2, 9), likely via looping out intervening sequences (8). These long-range enhancer–promoter interactions critically depend on, and are mediated by, self-interaction of LDB proteins (10, 11) through their N-terminal dimerization domain (DD) (12, 13).

Chip/LDB binds to a single-stranded DNA binding protein (SSDP, also known as single-stranded binding protein, SSBP) through the LDB1/Chip conserved domain (LCCD), a short region downstream of the DD (14, 15). The loss-of-function phenotypes of *Drosophila chip* mutants closely resemble those of *ssdp* mutants in various developmental contexts, although the former tend to be stronger and more pleiotropic than the latter (14, 16), especially in the early embryo (17). These phenotypic similarities indicate an intimate cooperation between Chip and SSDP in mediating enhancer function. Consistent with this, chicken Ssdp1 and Ssdp2 are required to confer transcriptionally active chromatin on loci controlled by Ldb1 (15), and murine Ssbp2 is required for hematopoietic stem cell maintenance, similarly to Lbd1 (18). The molecular basis underlying this intimate cooperation between Chip/LDB and SSDP is not known.

We recently discovered that Chip/LDB and SSDP form a stable complex called ChiLS (Chip/LDB-SSDP), which binds to NPFxD motifs within the nuclear Wnt signaling factor Pygopus (Pygo) and Osa/ARID1A (17), a chromatin-binding subunit of the BAF transcriptional coactivator complex (19). The Wnt enhanceosome is a ChiLS-containing multiprotein complex that is tethered to Wnt-responsive transcriptional enhancers via T cell factors/lymphoid-enhancer binding factors (TCF/LEF) and their associated Groucho/transducin-like enhancer (TLE) corepressors, to prime linked developmental control genes for timely Wnt responses (17, 20). The Wnt response of this complex is conferred by Pygo, which facilitates loading of the Wnt effector β-catenin via an adaptor called BCL9, thereby promoting transcriptional activation (ON state) (17, 20), while its rerepression (OFF state) appears to depend on Osa (21). The Wnt enhanceosome model envisages a pivotal role of ChiLS in the assembly and function of the complex, implicating ChiLS as its switch module: Its chromatin tethering involves a combination of enhancer-associated proteins (including LIM proteins, Pygo, TCF/LEF-associated Groucho/TLE ,and the BAF complex) that determine jointly the ON and OFF states of Wnt-controlled downstream genes (17, 20). Importantly, the stoichiometry of the minimal stable ChiLS complex is 2:4 (Chip/LDB:SSDP), as determined by size-exclusion chromatography followed by multiangle light-scattering (SEC-MALS) of purified recombinant proteins (17), implying that ChiLS contains 1 Chip/LDB dimer and 2 SSDP dimers.

Here, we report the crystal structures of the DD of SSDP and XLdb1, in the latter case with the help of DARPin (designed ankyrin repeat proteins) chaperones that mask a hydrophobic surface patch of the DD required for its SSDP binding. Systematic structure-led mutagenesis of conserved solvent-exposed residues of Chip/LDB and SSDP followed by in vitro and in vivo binding assays enabled us to generate an interaction map, and to derive a highly constrained structural model for ChiLS. Its rotationally symmetric SSDP_2_-LDB_2_-SSDP_2_ architecture with 2 predicted NPFxD-binding pockets on either side underscores its function as an integrating core module of the Wnt enhanceosome and other ChiLS-containing enhancer-binding complexes.

## Results

**Fig. 1.**
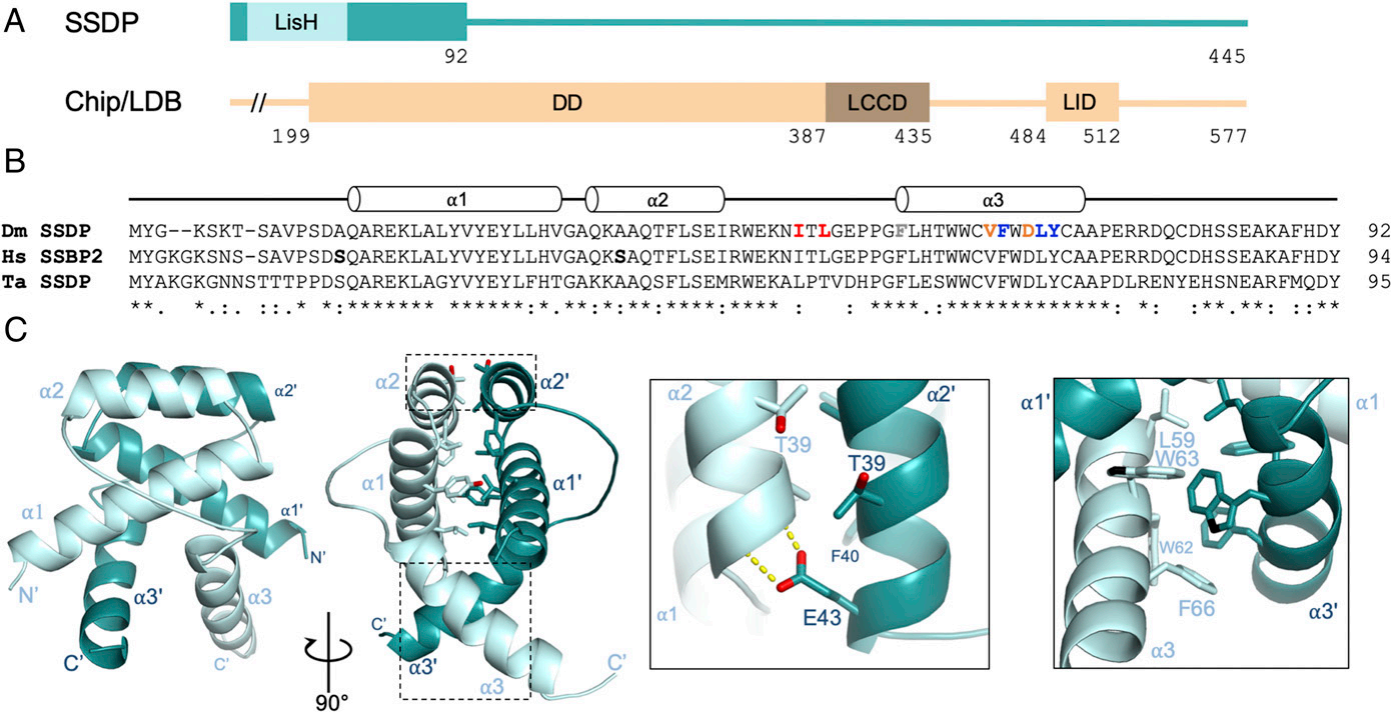
Structure of the SSDP dimer. (*A*) Cartoons of SSDP and Chip/LDB and their domains (numbers, *Drosophila* SSDP and Chip). (*B*) Sequence alignment of the N-terminal dimerization domain of *Drosophila* (Dm), human (Hs), and *Trichoplax adhaerens* (Ta) SSDP, with secondary elements indicated above; bold indicates amino acid variations between Hs SSBP2 and Dm SSDP (structured region only); colored indicates crucial mutated residues. (*C*) Ribbon representations of SSDP dimer, with helices labeled; (*Left Inset*) dimerization interface between α2 and α2′, with key residues shown in stick, and hydrogen bonds (between α2′ E43 and main chain α2 atoms) as yellow dashed lines; (*Right Inset*) α3/α3′ dimerization interface, with key hydrophobic residues shown in stick.

SSDP contains an N-terminal LisH domain known to form obligate dimers in other proteins (22), followed by a highly conserved extension and a long nonconserved C-terminal tail likely to be disordered (Fig. 1*A*). The extended N-terminal dimerization domain (called SSDP-N below; also known as the LUFS domain) (14) can form stable tetramers in vitro following bacterial expression (17). To determine the structure of SSDP-N, we optimized its boundaries and succeeded in obtaining crystals for SSDP_1–86_ that diffracted to 2.4 Å. As we were unable to use molecular replacement with the LisH domain as a search model, we generated 5 different selenomethionine (SeMet)-labeled mutants, 1 of which yielded diffracting crystals that enabled us to determine the structure of SSDP-N (*SI Appendix*, Table S1).

### Structure of the SSDP-N Dimer.

The asymmetric crystallographic unit contains a classic LisH dimer-fold in which 2 α-helices (α1, α2) (Fig. 1*B*) associate to form an antiparallel 4-helix bundle (22), the hydrophobic interface of which is strengthened by additional hydrogen bonds in its periphery (Fig. 1*C*). A third helix (α3) interacts with its counterpart (α3′) from the opposite dimer to form an X-shaped structure, tucked under the 4-helix bundle. It thus resembles TBL1, a subunit of a transcriptional corepressor complex and the only other LisH-containing protein known to form tetramers (23), except that the lengths and angles between α3 and α3′ differ in the 2 structures (*SI Appendix*, Fig. S1). As in other LisH dimers, the dimer interface is extensive (burying ∼2,500 Å^2^), which explains why SSDP forms obligate dimers, and why SSDP monomers are not detectable (17) (see also below). Indeed, the *K*_d_ for LisH-mediated self-association of Lis1 was reported to be subfemtomolar (22).

**Fig. 2.**
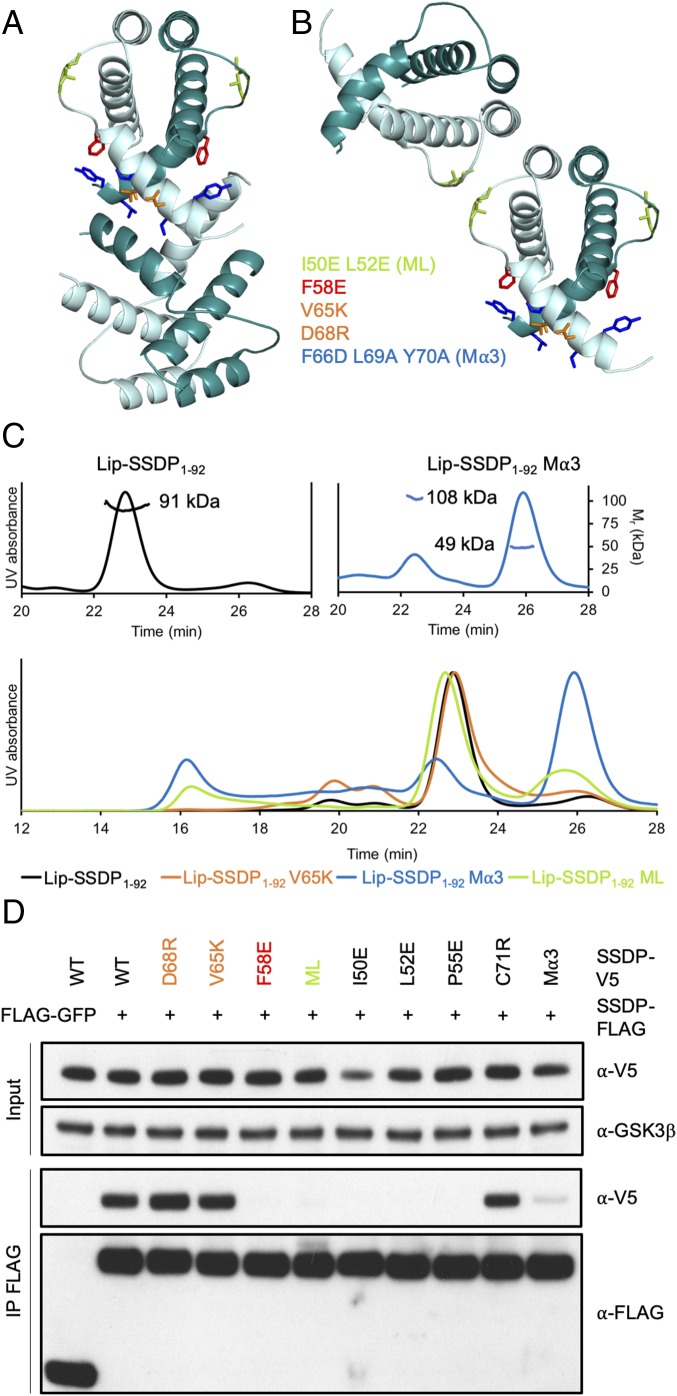
Two modes of SSDP tetramerization. (*A* and *B*) Tetramerization of SSDP through (*A*) α3 interface or (*B*) loop interface (between α2 and α3), with key mutated residues in stick (same color in all panels). (*C*) SEC-MALS profiles of WT and selected Lip-SSDP_1–92_ mutants, as indicated; numbers in *Upper*, M_r_ determined by MALS (corresponding to SSDP_2_ and SSDP_4_; expected M_r_, 47 and 94 kDa, respectively). (*D*) co-IP assays of selected SSDP mutants in transfected LDB1/2 DKO cells (see also *SI Appendix*, Figs. S2 and S3).

Within the crystal lattice, symmetry-related dimers associate via 2 perpendicular pairs of antiparallel α3 helices. The resulting interlocked bundle of 4 α-helices bury ∼1,400 Å^2^ (Fig. 2*A*), and is held together entirely via hydrophobic contacts, with valine 65 (V65) occupying a central position in the tetramer interface (“α3 interface,” below). The same tetramer was observed for human SSBP2 (whose structured region differs from fly SSDP-N only by 2 semiconserved residues) (Fig. 1*B*) (24) whose structure was reported during the final stages of our work. Intriguingly, some of the hydrophobic residues in the loop between α2 and α3 also mediate association between symmetry-related dimers in the crystal, resulting in alternative tetramer configurations (Fig. 2*B*). Although these loop-mediated interactions bury smaller interfaces (602 Å^2^ and 624 Å^2^, respectively; ”loop interface,” below), they proved to be functionally relevant in vivo (see below). We note that TBL1 tetramerizes via α2 (*SI Appendix*, Fig. S1) via residues that are not conserved in SSDP.

### Alternative Modes of Self-Interaction of SSDP Dimers.

To test the functional relevance of the different modes of SSDP self-interactions, we designed repelling amino acid substitutions in solvent-exposed hydrophobic residues in the 2 alternative interfaces of SSDP-N, mutating a centrally located valine in the α3 interface (V65K), and 2 hydrophobic residues in the loop interface (I50E L52E, called ML) that mediate mutual interactions in the crystals (Fig. 1*B*). We also included a previously designed triple mutation of hydrophobic residues flanking V65 (F66E L69A Y70A, called Mα3) that blocks SSBP2-N tetramerization (24). These mutations were introduced into Lip-SSDP_1–92_ and their self-interaction was tested by SEC-MALS. As expected (24), Mα3 blocks tetramerization of SSDP-N, but V65K does not (Fig. 2*C*), likely because this single mutation is too weak to do so, given the extensive α3 interface. ML also reduces tetramerization of SSDP-N significantly (Fig. 2*C*). We conclude that both modes of SSDP-N tetramerization can occur, with α3 being the preferred interface. Tetramerization likely occurs during bacterial expression since SSDP-N dimers and tetramers are stable in solution for >8 h, and do not interconvert following purification.

Next, we designed another 16 substitutions in conserved solvent-exposed residues that should not perturb the fold of SSDP-N (*SI Appendix*, Fig. S2*A*), and introduced these (plus V65K) into full-length V5-tagged SSDP. We then coexpressed these with WT FLAG-tagged SSDP in HEK293T cells from which we deleted LDB1 and LDB2 by CRISPR engineering (below, double-knockout [DKO] cells) (*SI Appendix*, Fig. S3), to test their mutual association by coimmunoprecipitation (co-IP) in the absence of endogenous LDB. As expected, SSDP-FLAG co-IPs efficiently with WT and most mutant SSDP-V5, except for I50E, L52E, P55E, and F58E (each altering the loop surface) (Fig. 2*B* and *SI Appendix*, Fig. S2*A*), which do not coimmunoprecipitate at all. Mα3 produces a reduced co-IP signal, partly because this triple-mutant is somewhat unstable in cells (Fig. 2*D* and *SI Appendix*, Fig. S2*B*). We conclude that full-length SSDP dimers also self-associate in cells upon overexpression, but do so entirely through their loop interface. This implies that the SSDP C terminus blocks self-association of full-length SSDP via its α3 surface.

### Structure of the LDB Dimer.

Next, we purified fragments spanning the DD or DD-LCCD from human LDB1, linked to a cleavable His_6_ and Lipoyl (Lip) tag, but could not crystallize these. Screening LDB orthologs from different species, we found that *Xenopus laevis* Ldb1 (XLdb1) (1) yielded the most stable protein after removal of solubility tags. XLdb1 is closely related to human LDB1 (*SI Appendix*, Fig. S4), but we did not succeed in obtaining diffracting crystals with this protein either, largely because the DD and DD-LCCD undergo nonspecific aggregation over time. We therefore resorted to chaperone-based approaches, selecting nanobodies (25) or high-affinity DARPins, genetically engineered antibody-mimetics based on consensus ankyrin repeat proteins and selected from diverse synthetic libraries (26), against the purified DD or ChiLS complex (*SI Appendix*, *Supplementary Methods*). None of 35 selected nanobodies facilitated crystallization, but we obtained DD crystals with 5 of 15 selected DARPins (*SI Appendix*, Fig. S5) that diffracted to 2.0 to 2.59 Å (*SI Appendix*, Table S1), depending on the DARPin (*SI Appendix*, Fig. S6*A*). None of these 5 DARPins bind to the DD-LCCD nor to the assembled ChiLS complex.

Next, we determined the crystal structures of the DD (XLdb1 20-200) bound to DARPin2, -3, -5, -7, or -10 (*SI Appendix*, Fig. S6). Remarkably, each of the 5 DARPins (despite their different sequences) recognizes the same lateral surface patch of the DD, perpendicular to its dimerization interface and facing outwards (*SI Appendix*, Fig. S6*B*). This patch is highly hydrophobic (e.g., Y81, I83, and L87 engage in direct DARPin contacts), which explains why its masking by DARPins was crucial to prevent nonspecific aggregation of the DD via this patch (called “hydrophobic patch,” below).

**Fig. 3.**
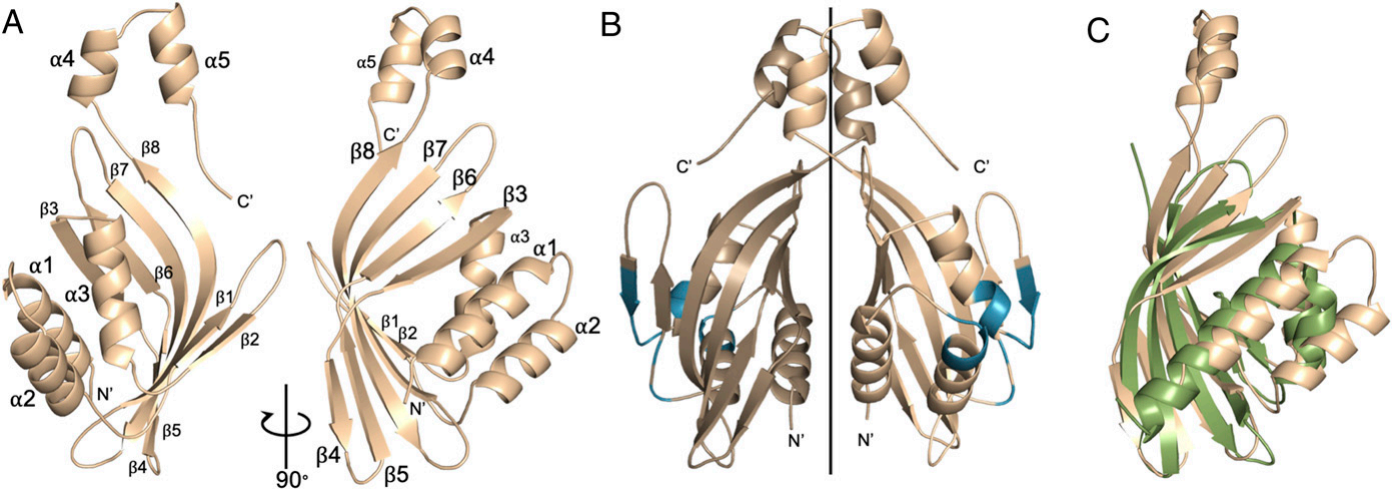
Structure of the DD. (*A* and *B*) Ribbon representations of (*A*) the DD monomer (with α-helices labeled) and (*B*) the DD dimer; vertical line is the symmetry axis; blue is the DARPin-binding patch. (*C*) Superimposition of the DD (wheat) and NTF2 (green; 1JB5) (see also *SI Appendix*, Fig. S7).

The DD adopts a cone-shaped α+β barrel-fold, composed of a long, highly curved antiparallel β-sheet (formed by β1 to 6) whose cavity is filled by 3 α-helices (α1 to α3) that run roughly parallel to the β-sheet (Fig. 3*A*). This structure is capped by a short C-terminal extension at the apex of the DD, formed by 2 intertwined α-helices (α4 and α5) that contribute to the dimer interface (Fig. 3*B*). The whole dimer interface is extensive (burying ∼1,500 Å^2^), which explains why the DD dimer is highly stable in solution.

Curiously, the closest structural relative of the DD is scytalone dehydratase (27) (rmsd ∼2 Å), a bacterial ketosteroid isomerase (*SI Appendix*, Fig. S7*A*). The DD also resembles the fold of other members of this enzyme family (28), which are among the most efficient enzymes known (29). The DD fold is also found in eukaryotes; for example, in nuclear transport factor-2 (NTF-2) (30) (rmsd ∼2.2 Å) (Fig. 3*C*). Whether any of these structural similarities have functional significance is unclear. The oligomeric state of the DD folds is variable, ranging from monomeric to tetrameric, and their dimerization modes also vary, with some folds dimerizing via their curved β-sheets (*SI Appendix*, Fig. S7*B*). However, none of the known DD folds contain an intertwined apical extension, as seen in the XLdb1 DD (Fig. 3*B* and *SI Appendix*, Fig. S7*B*). This apex may help to determine the dimerization mode of the DD, and stabilize the Chip/LDB dimer, given that a region spanning this apex is essential for β-globin transcription during erythroid maturation (albeit not for LDB1 dimerization per se, as the DD core may be partially competent to dimerize by itself) (11).

### Binding Between Chip/LDB and SSDP.

Next, we attempted to crystallize the ChiLS complex, using bicistronic coexpression of SSDP_1–92_ and DD-LCCD from XLdb1. LCCD is a conserved 49-amino acid stretch (Fig. 1*A* and *SI Appendix*, Fig. S4) required for binding to SSDP (14, 15). It spans 2 predicted α-helices (α6 and α7), whereby α6 is separated from the DD by a linker of 9 conserved residues (*SI Appendix*, Fig. S4). We designed 2 DD fragments with C-terminal extensions, namely DD-LCCD1 (20-244, spanning α6 and α7) or DD-LCCD2 (20-226, spanning only α6). Both form a stable complex with SSDP_1–92_, implying that α6 suffices for SSDP binding at high protein concentrations during bacterial expression. We purified both complexes, but did not obtain diffracting crystals despite testing a wide range of conditions, as well as cocrystallization with nanobodies or DARPins.

**Fig. 4.**
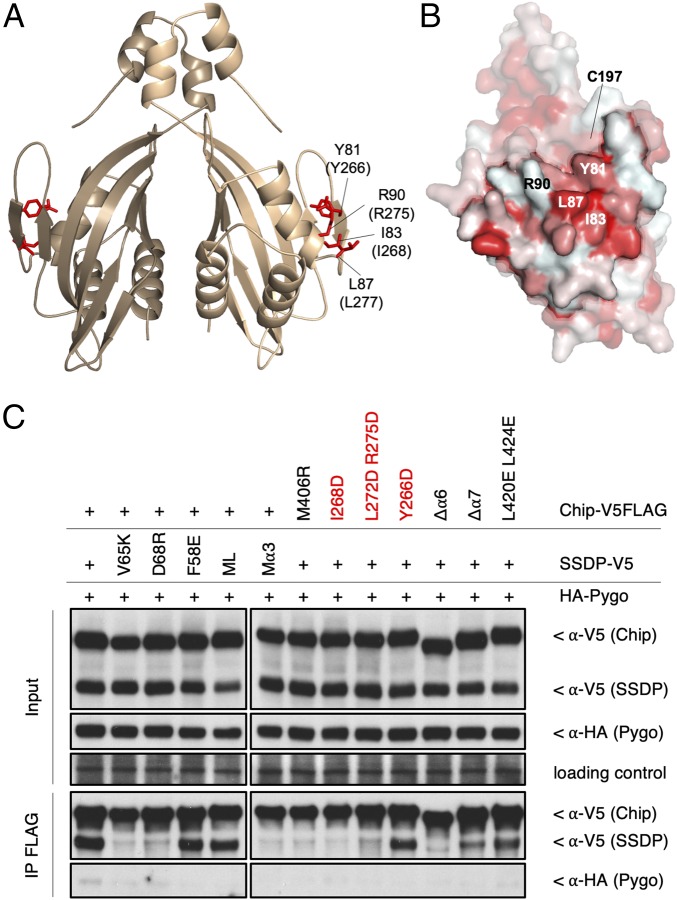
Mutual interactions between Chip/LDB, SSDP, and Pygo. (*A*) Functionally relevant solvent-exposed residues in the DD (Chip residue numbers in parenthesis), tested for interaction with SSDP and Pygo. (*B*) Surface representation of DD, with hydrophobic patch mediating interaction with SSDP highlighted; Y81, L87, and R90, DARPin-binding residues; C197 marks the C terminus of the structured part of the DD, and start of LCCD. (*C*) co-IP assays of selected mutants in transfected HEK293T cells (see also *SI Appendix*, Table S3).

We therefore took an alternative approach, namely systematic mutational analysis of conserved solvent-exposed residues of each ChiLS dimer subunit (Fig. 4*A*), to identify their surfaces required for mutual interaction. Since DARPin binding to their cognate patch of the DD (Fig. 3*B*) blocks its interaction with SSDP, we initially focused on this hydrophobic patch, designing a set of repelling mutations (*SI Appendix*, Fig. S8), which might block binding between DD-LCCD1 and SSDP. Indeed, 2 of them do so (L87D R90D and Y81D L87D R90D) (*SI Appendix*, Table S2). Since Y81, L87, and R90 are direct DARPin-interacting residues (Fig. 4*B*), this implicates the hydrophobic patch in the interaction between SSDP and the DD.

Next, we designed additional mutations in the apical, lateral, and basal surfaces of the DD dimer, in LCCD and its linker to DD (*SI Appendix*, Fig. S8), and we also generated internal deletions that remove LCCD α6 or α7 (Δα6, Δα7), for co-IP assays in transfected HEK293T cells aimed at testing the binding between full-length proteins. To minimize cross-reaction with endogenous LDB, we introduced these mutations into Chip-V5FLAG, and coexpressed them with SSDP-V5. This confirmed the functional importance of Y81, L87, and R90, but also identified additional residues near the hydrophobic patch as critical for co-IP with SSDP (Fig. 4*C* and *SI Appendix*, Table S3), marking a contiguous hydrophobic surface in the DD (Fig. 4*B*). In contrast, repelling point mutations in the apical or basal surface of the DD did not affect co-IP between Chip and SSDP (*SI Appendix*, Table S3). We conclude that a lateral hydrophobic surface patch of the DD is crucial for its association with SSDP. This implies that a single Chip/LDB dimer can accommodate 2 SSDP dimers.

In addition to this hydrophobic patch, we also found that α6 is crucial for co-IP between Chip and SSDP, while Δα7 also reduces it; indeed, mutation of a single methionine in α6 (M406R) reduces co-IP between the 2 proteins to background levels (Fig. 4*C*). M402E also blocks co-IP, and L405D reduces it, as does a double mutation in α7; however, none of the mutations in the conserved linker between the DD and LCCD affect co-IP (*SI Appendix*, Table S3). Thus, α6 is essential for binding between Chip and SSDP, and M402 and M406 may directly contact SSDP. Consistent with this, a 10-amino acid stretch spanning these methionines in Chip is essential for its binding to SSDP (14), as is a similar 6-amino acid stretch in chicken Ldb1, whereby the latter is also critical for motoneuron specification in the chicken embryo (15), indicating the physiological importance of LCCD α6. Either α6 constitutes the SSDP-binding surface of Chip/LDB, together with the lateral hydrophobic patch, or this patch binds primarily to α6, which in turn binds to SSDP, perhaps the more likely scenario, for reasons discussed below. In contrast, α7 appears to be auxiliary in mediating the Chip/LDB-SSDP interaction.

To define the Chip-binding surface of SSDP, we conducted co-IP assays between WT Chip-V5FLAG and our panel of 17 SSDP-V5 mutants (Fig. 2*D* and *SI Appendix*, Fig. S2*A*), following coexpression in HEK293T cells. Most SSDP mutations have no effect (including ML), but D68R reduces co-IP, as does Mα3, while V65K essentially blocks co-IP (Fig. 4*C*). We conclude that SSDP binds to Chip/LDB via its α3 surface (Fig. 2*A*).

### Dependence of Pygo Binding on ChiLS Complex Assembly.

Next, we tested our SSDP and Chip mutants for interaction with *Drosophila* Pygo upon coexpression in HEK293T cells. This interaction is relatively weak, and not easily detectable by co-IP (17). However, we consistently obtained a co-IP signal between Pygo and WT Chip and SSDP, but with none of the mutants that block the Chip–SSDP interaction (Fig. 4*C* and *SI Appendix*, Table S3). This suggests that the integrity of the ChiLS complex is essential for Pygo binding, consistent with our previous evidence that peptides spanning NPFxD from Pygo or human Pygo2 bind to the assembled recombinant ChiLS complex but not to its subunits alone (17). Notably, F58E blocks Pygo binding to ChiLS while it barely affects binding between Chip and SSDP, which suggests that SSDP F58 contributes critically to the NPFxD-binding pocket of ChiLS.

To corroborate our co-IP results with in vitro binding assays between recombinant proteins, we used NMR spectroscopy (17), probing an ^15^N-labeled 27 amino acid peptide spanning NPFED from human Pygo2 (^15^N-Lip-NPFED_Pygo2_) with purified recombinant ChiLS. This revealed that WT ChiLS interacts with ^15^N-Lip-NPFED_Pygo2_, even without LCCD α7 (*SI Appendix*, Fig. S9), indicating that the latter is dispensable for ChiLS binding to NPFxD at high protein concentration. We also used this assay to determine whether excess unlabeled Lip-NPFDD peptide from Pygo can compete for binding of purified ChiLS to an ^15^N-labeled 26 amino acid peptide from Osa (^15^N-Lip-NPFED_Osa_), which was the case (*SI Appendix*, Fig. S10). This implies that the same ChiLS pocket can accommodate the NPFxD motif of either ChiLS ligand.

### A Highly Constrained Structural Model of ChiLS.

**Fig. 5.**
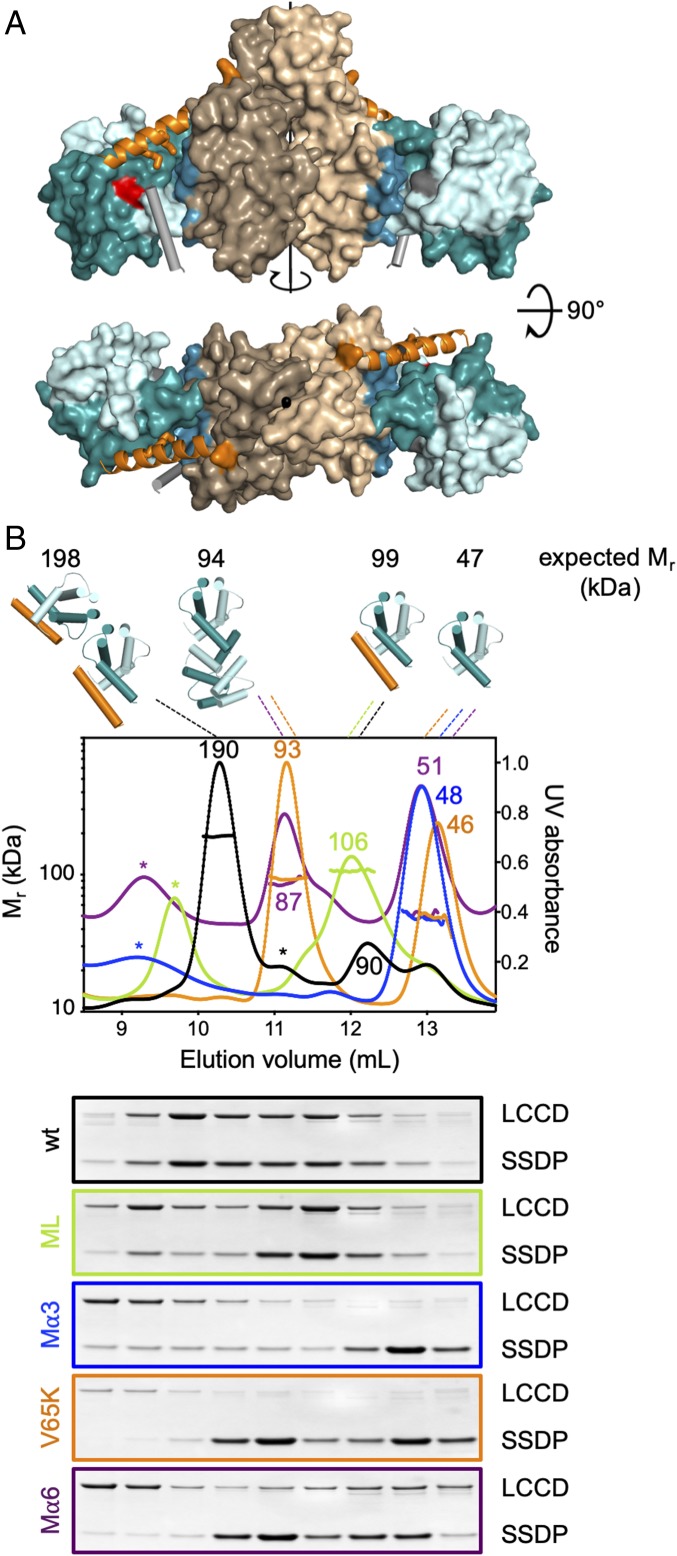
Structural model of the ChiLS complex. (*A*) Structural model of ChiLS, derived from stoichiometry of complex and mutational analysis (see text); (*Upper*) side view; (*Lower*) view from top, revealing rotational symmetry of ChiLS and its binding sites for SSDP and NPFxD (whose precise positions and angles relative to the DD are arbitrary in this model). SSDP dimers (cyan) were docked manually onto the DARPin-binding patch (blue) in each lateral surface of the DD (wheat); (orange) predicted LCCD α6, with SSDP-binding residues (M402, M406) in stick; positions of the pockets for NPFxD (gray rods) are restrained by proximity to LCCD α6 (orange) and SSDP F58 (red); note that SSDP′ F58 (gray) differs from SSDP F58 (red) regarding its structural environment. (*B*) SEC-MALS profiles of complexes (cartoons above panels) formed between coexpressed WT and mutant MBP-LCCD_Chip_ and Lip-SSDP_1–92_; numbers are M_r_ values determined by MALS (within panels), or as expected (above panels); asterisks are large aggregates; below, PAGE revealing proteins in corresponding preparative SEC fractions.

Next, we constructed a model of the ChiLS complex, taking into account its constituent dimer structures (Figs. 1*C* and 3*B*), the results from our mutational analysis (Fig. 4 and *SI Appendix*, Tables S2 and S3) and the previously determined 4:2 (SSDP:Chip/LDB) stoichiometry of the ChiLS complex (17). The only ChiLS configuration that is consistent with all our data corresponds to an SSDP_2_-LDB_2_-SSDP_2_ architecture with rotational symmetry (Fig. 5*A*). In this model, the lateral hydrophobic surface patches of each DD subunit (Fig. 5*A*, blue) interface with their downstream-adjacent LCCD α6 (Fig. 5*A*, orange) and the α3 surface of an SSDP dimer (Fig. 5*A*, dark cyan). Because of its inherent rotational symmetry, the complex can accommodate two NPFxD ligands (Fig. 5*A*, gray rods), through opposite pockets whose positions are defined by proximity to the LCCD α6 methionines (Fig. 5*A*, orange sticks) and SSDP F58 (Fig. 5*A*, red patch).

To corroborate our model, we asked whether a minimal LCCD fragment could bind SSDP, and if so, whether this would be sensitive to mutations in its α3 or loop surface. We therefore conducted SEC-MALS of Chip-LCCD (residues 384 to 436, of which 47 are identical in human LDB1) (*SI Appendix*, Fig. S4) tagged with maltose binding protein (MBP) coexpressed with WT, V65K, Mα3, or ML Lip-SSDP_1–92_. As a further control, we also included a triple mutant of LCCD (M402E L405D M406R, Mα6) expected to block binding to SSDP (Fig. 4*C*). Indeed, the WT proteins form predominantly a single complex eluting in 1 main SEC peak, containing similar amounts of LCCD and SSDP as judged by PAGE of the corresponding fractions (Fig. 5*B*). Based on its molar mass (M_r_ = 190 kDa) as determined by MALS, the complex in this peak most likely corresponds to LCCD-SSDP_4_-LCCD: That is, 2 LCCD-SSDP_2_ complexes interacting via the SSDP loop interface (Fig. 5*B*, cartoon). We also observed a smaller peak (M_r_ = 90 kDa), likely corresponding to LCCD-SSDP_2_, in addition to minor peaks with considerably higher molar masses, likely corresponding to unspecific aggregates (Fig. 5*B*, asterisks). As expected, none of the mutants show the 190-kDa complex: SSDP Mα3 neither binds to LCCD nor tetramerizes, and so only forms dimers; SSDP V65K and LCCD Mα6 cannot bind to their partner subunits, and so the 2 main peaks observed with these mutants correspond to LCCD and SSDP_4_ (Fig. 5*B*, cartoons). Importantly, SSDP ML can interact with LCCD through α3, and so forms LCCD-SSDP_2_ (possibly in addition to coeluting SSDP_4_) (Fig. 5*B*). This confirms that 1) LCCD can bind to SSDP directly via α6, and 2) SSDP dimers can undergo mutual interactions via their loop surfaces if bound to LCCD. Thus, in the presence of LCCD, the α3 surface of SSDP prefers to bind to LCCD rather than itself.

## Discussion

Our work has led to a highly constrained structural model of ChiLS, the core complex of the Wnt enhanceosome (17, 20). Its rotational symmetry implies that ChiLS contains 2 structurally identical NPFxD-binding pockets, each bordered by an SSDP dimer and LCCD α6 (and possibly DD residues) (Fig. 5*A*), and each binding either Pygo2 or Osa NPFxD (*SI Appendix*, Fig. S10). Thus, 1 single ChiLS core complex, via its 2 NPFxD pockets, could accommodate Pygo as well as the Osa/ARID1 subunit of the BAF complex simultaneously. This is consistent with the notion that Pygo and the BAF complex are constitutive components of the Wnt enhanceosome (20).

ChiLS is also found in other enhancer-binding complexes, most notably those binding to the remote LCR enhancer that controls β-globin genes during erythroid maturation (8, 18, 31), but also to transcriptional enhancers of developmental control genes (1–4), and of genes that control stem cell maintenance (5, 6) and normal as well as malignant erythroid differentiation (7, 32, 33). N-terminal domains of Chip/LDB proteins self-associate (12, 13), and this property is crucial for mediating long-range interactions between remote enhancers and proximal promoters (2, 8, 10, 11). The DD dimer, as revealed by our structural analysis (Fig. 3*B*), provides the molecular basis for this function of Chip/LDB in mediating long-range enhancer–promoter interactions. From its structure, it is difficult to see how DD could oligomerize, as previously proposed (2, 8, 13), but we note that recombinant DD has a tendency aggregate in vitro, likely via its hydrophobic DARPin-binding patch.

Recombinant SSDP-N clearly tetramerizes via its α3 surface (24) (Fig. 2 *A* and *C*). However, in our co-IP assays involving expression of full-length proteins in cells, SSDP uses its α3 surface exclusively for Chip/LDB binding, while it self-associates via its loop surface (Fig. 2*C*). Furthermore, our evidence suggests that LCCD binding to SSDP may promote loop-mediated self-association of SSDP (Fig. 5*B*), possibly by inducing a conformational change of its loop surface. Indeed, Chip/LDB-bound SSDP dimers that self-associate via their loop interfaces (Fig. 2 *B*–*D*) could promote dimerization of the core ChiLS complex (Fig. 5*B*), assembling higher-order oligomers that could be instrumental for the function of ChiLS in mediating long-range enhancer–promoter contacts. Whatever the case, our results strongly support the notion that SSDP is essential for the function of Chip/LDB proteins in transcriptional activation by remote enhancers (14, 17, 18, 31, 34, 35).

Our structural model of ChiLS provides mechanistic insight into how ChiLS-containing enhancer complexes integrate multiple inputs from signaling and lineage factors: Because of its symmetrical SSDP_2_-Chip/LDB_2_-SSDP_2_ architecture, a single ChiLS core complex can bind simultaneously to 2 different NPFxD ligands (17, 20) (Fig. 5*A*) and to 2 sets of distinct enhancer-binding proteins via its LID (binding to LIM-containing proteins, or GATA and bHLH factors) (3, 4, 7, 8). Therefore, ChiLS is uniquely poised as an integrating core module of multiprotein complexes that are tethered to transcriptional enhancers by specific combinations of DNA-binding proteins and their associated signal-responsive cofactors. Furthermore, by exchanging some of these factors, ChiLS can switch enhancer complexes between ON and OFF states.

## Materials and Methods

### Plasmids.

Plasmids used for cell-based assays and bacterial expression were used as described previously (17), including His_8_ (for DARPins), His_6_-Lip (for XLdb1 and SSDP mutants), and bicistronic expression vectors (for coexpression of SSDP_1–92_ and MBP-LCCD).

### Protein Purification.

Proteins were expressed in BL21(DE3)-RIL, and purified with Ni-NTA resin followed by gel filtration. For crystallization, the solubility tags were removed by gel filtration following digestion with tobacco etch virus protease.

### Functional Assays in Human Cells.

HEK293T cells were grown in DMEM, supplemented with 10% FBS and transfected in 6-well plates with PEI. One-hundred nanograms per well of Chip, 400 ng per well of SSDP, and 500 ng per well of Pygo constructs were used for all transfections.

### DARPin Selection.

Ribosome display selections against the DD or ChiLS complex were carried out as detailed in *SI Appendix*, *Supplementary Methods*.

### NMR Spectroscopy.

Proteins were expressed in minimal medium supplemented by ^15^N-ammonium chloride and purified as described above. NMR spectra were recorded with a Bruker Avance III spectrometer at 600 MHz ^1^H, as described previously (17).

### SEC-MALS.

Purified proteins were analyzed with an Agilent 1200 Series chromatography system connected to a Dawn Heleos II 18-angle light-scattering detector combined with an Optilab rEX differential refractometer (Wyatt). Samples were loaded onto a Superdex-200 10/300 gel-filtration column (GE Healthcare) at 2 mg/mL and run at 0.5 mL/min in buffer (PBS, 1 mM DTT). Data were processed with Astra V software.

### Crystallization.

Concentrated proteins (10 to 20 mg/mL) were used for initial screens with ∼1,500 different crystallization conditions in 100 + 100-nL drops in a 96-well sitting-drop format. Crystals emerged under multiple conditions (*SI Appendix*, Table S1) after growing for several days at 19 °C by the vapor-diffusion method, and were directly flash-frozen in liquid nitrogen. For determination of structures, see *SI Appendix*, *Supplementary Methods*.

## Supplementary Material

Supplementary File
