# Supplementary material for: Rotational symmetry of the structured Chip/LDB-SSDP core module of the Wnt enhanceosome
Source: Proc Natl Acad Sci U S A. 2019 Sep 30;116(42):20977–83. doi: 10.1073/pnas.1912705116 (PMC6800368; doi:10.1073/pnas.1912705116)
Supplement: Supplementary File [file pnas.1912705116.sapp.pdf]

PNAS
www.pnas.org

Supplementary Information for

Rotational symmetry of the structured Chip/LDB-SSDP core module of the Wnt enhanceosome

Miha Renko, Marc Fiedler, Trevor J. Rutherford, Jonas Schaefer, Andreas Plückthun & Mariann Bienz

Email: mb2@mrc-lmb.cam.ac.uk

This PDF file includes:

Supplementary Methods
Figs. S1 to S10
Tables S1 to S3
Supplementary References

www.pnas.org/cgi/doi/10.1073/pnas.1912705116

Supplementary Methods

Generation of DARPin binders against ChiLS - To generate suitable DARPin binders, either only biotinylated Lip-DD (via the Avi-tag present at its N-terminus) or Lip-DD-LCCD1-SSDP (via biotinylated DD) were immobilized on magnetic streptavidin beads and used in DARPin selections by Ribosome Display [1,2]. In each panning round, the target concentration presented was decreased while the washing stringency was simultaneously increased to enrich for binders with high affinities. This enrichment was further enhanced by off-rate selections employing non-biotinylated targets [3]. Finally, also negative selections against an Avi-tagged Lip-construct were performed to direct selected binders to DD.

After four rounds of selection, the enriched pool was cloned into a bacterial pQIq-based expression vector, allowing the production of both N-terminally His_8- and C-terminally FLAG-tagged DARPins. Nearly 400 colonies of transformed *Escherichia coli* XL1Blue were picked for each selection and the encoded DARPins expressed at small scale. Bacterial crude extracts were subsequently used in Homogeneous Time Resolved Fluorescence (HTRF) based screenings, detecting the binding of the FLAG-tagged candidate DARPins to streptavidin-immobilized targets by measuring FRET signals between labeled streptavidin and anti-FLAG antibodies. Roughly, 40% of analyzed candidates showed clear signals in these initial HTRF screens and 32 candidates of each selection with different signal intensities were sequenced. Subsequently, a total of 43 clones with complete and unique sequences were expressed in larger scale and Ni-NTA purified for SEC analyses. Of those DARPins tested, 33 showed predominantly monomeric behavior. Based on results of pull-downs with Lip-DD-LCCD1-SSDP and Lip-DD, 15 DARPins were chosen and recloned into a pQIq-based expression vector for the production of constructs, containing an N-terminally His_{10}-HA-tag which can be cleaved-off after purification by 3C precision protease to yield untagged DARPins for co-crystallization. The sequences of those DARPins are shown in Fig. S4.

Determination of crystal structures - The initial SSDP structure was determined by single anomalous dispersion using SeMet-labeled crystals. For DD-Darpin3, initial preliminary phase information was obtained by molecular replacement, using the structure of DARPin as a search model. The structure of DD was then built iteratively, using ArpWarp and manual interventions. SeMet-labeled DD-DARPin10 was also used for building a structural model based on SeMet-derived phase information. Both approaches yielded identical results.
Determination of the heavy atom substructure and initial CA-model building were done using SHELXD[4]. The structure was built with ArpWarp[5] and manually edited subsequently with COOT [6]. All structures were refined with REFMAC5[7] and deposited at the PDB.

Isolation of LDB1/2 double-knockout cell line - To generate LDB1/2 DKO cells, HEK293T cells were initially transfected with plasmid pX458 (AddGene catalog number 48138) encoding Cas9 and guide RNAs targeting genomic loci of LDB1[8]. Clones grown from single cells were screened by genomic DNA sequencing and deletions were confirmed by immunoblotting with anti-LDB1 antibodies (OriGene, catalog number

TA308742). In the next step, LDB1/2 DKO were generated from LDB1 KO cells following the same procedure. The gRNA sequences, amplification and sequencing primers are shown in Fig. S3.

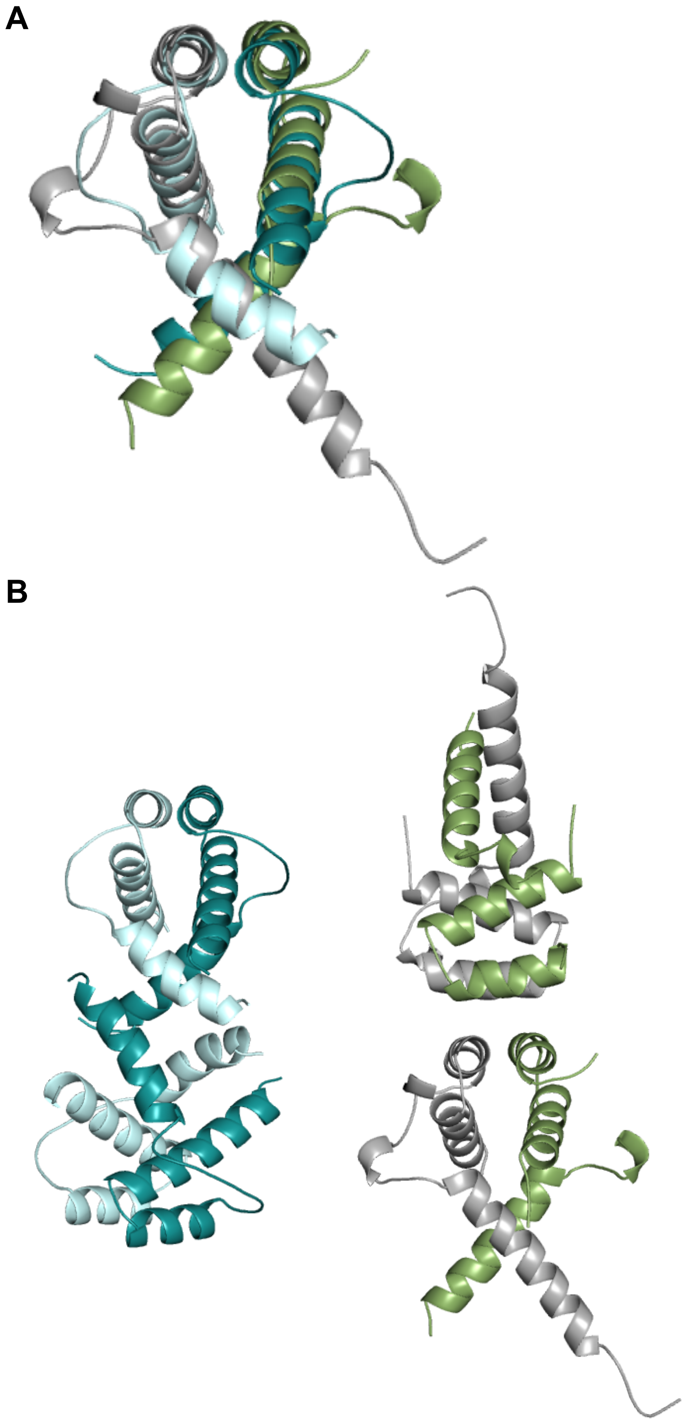

Fig. S1 Comparison between SSDP and TBL1 structures
(**A**) Superimposition of SSDP (*cyan*) and TBL1 (2XTC; *green*, *grey*) dimers. (**B**) Different modes of tetramerization; *left*, SSDP tetramerization via α3; *right*, TBL1 tetramerization via α2.

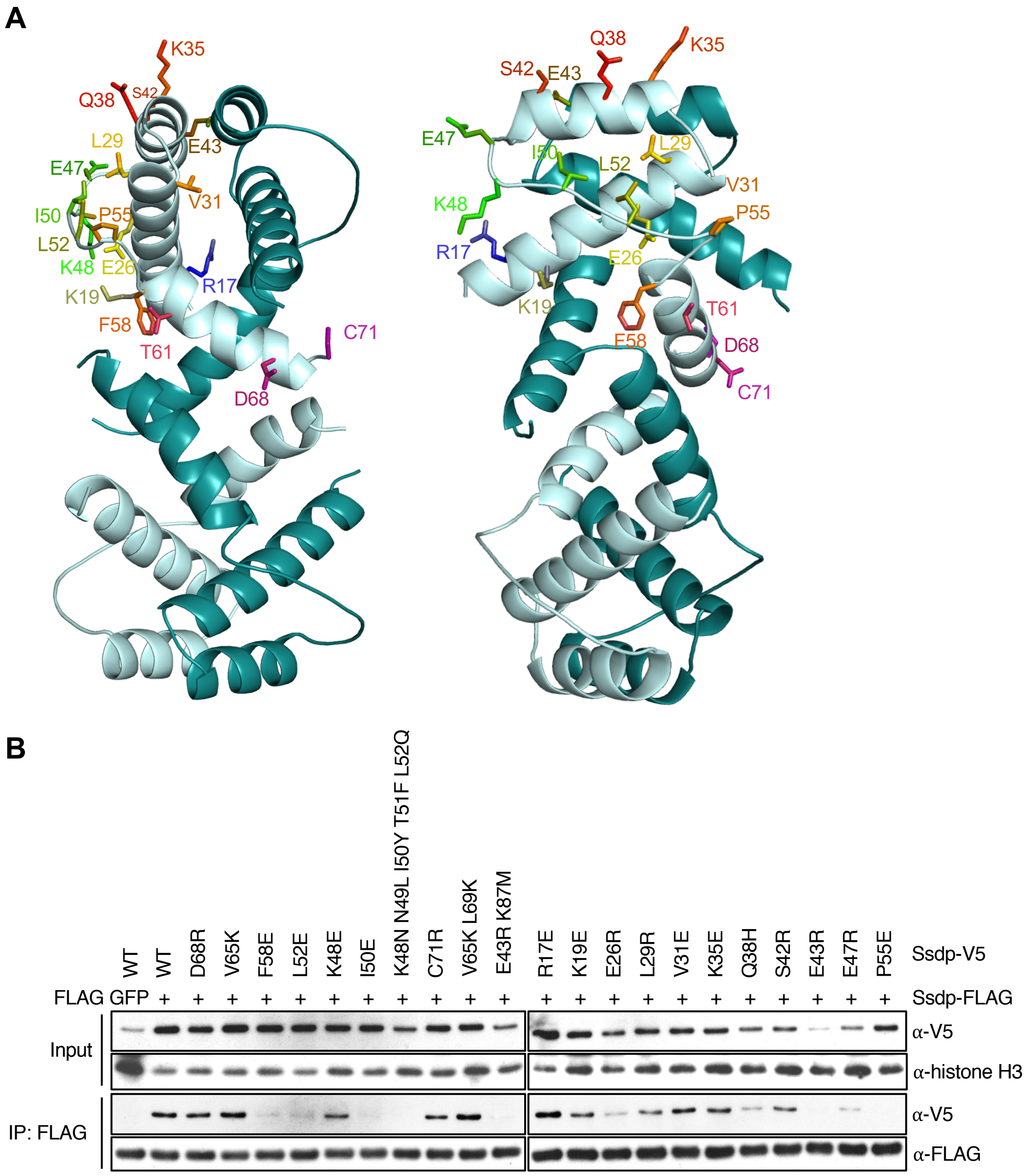

Fig. S2 Self-interaction of SSDP dimers in cell-based assays

(**A**) Position of 16 substitutions in solvent-exposed residues in SSDP, tested for SSDP coIP in cell-based assays. (**B**) coIP of SSDP upon co-expression in HEK293T cells.

A

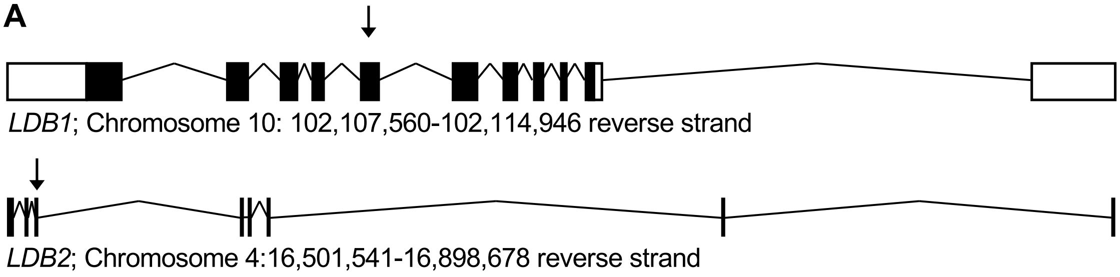

B

sgRNAs

LDB1 g1	G AAGATGCTGCGGAAGTAGCG
LDB1 g2	GCGGAAGTAGCGTGGGATCA
LDB2 g1	GGACTGCGACCAGTGTACCA
LDB2 g2	G CTGGTCGCAGTCCACCGTGA

Amplification primers

LDB1 F2	TCTCCCCATGTGGCTAGTGA
LDB1 R1	AGGCACACACCATATGGCAA
LDB2 F4	CTGACCACGCGAAAATAGGA
LDB2 R1	ACATGACTGGGTAACTGGCC

Sequencing primers

LDB1 F3	TATGCCTCCCTGGGGAACAG
LDB2 F5	GGCACTAGAACCTCTCTTCC

C

Clone Name	LDB1 A3	LDB1 A6	LDB1 B13	LDB1/2 D4	LDB1/2 D7	LDB1/2 D9	LDB1/2 D19
Parental line	HEK 293T	HEK 293T	HEK 293T	LDB1 A3	LDB1 A3	LDB1 A3	LDB1 B13
sgRNA used	LDB1 g1	LDB1 g1	LDB1 g2	LDB2 g1	LDB2 g2	LDB2 g2	LDB2 g1
Sequencing	null	null	null	null	null	null	null

D

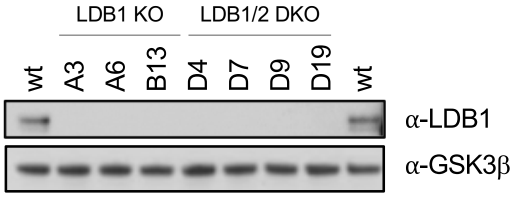

Fig. S3 Isolation and characterication of LDB1/2 DKO cell line
(A) Chromosomal organization of *LDB1* and *LDB2*. Arrows indicate targeted exons. **(B)** Sequences of gRNAs, amplification and sequencing primers used. **(C)** Overview of generated cell lines. Genomic sequencing confirmed that both *LDB1* and *LDB2* were disrupted in each DKO line. **(D)** Western blots of lysates from LDB1 KO and LDB1/2 DKO, probed with antibody against LDB1.

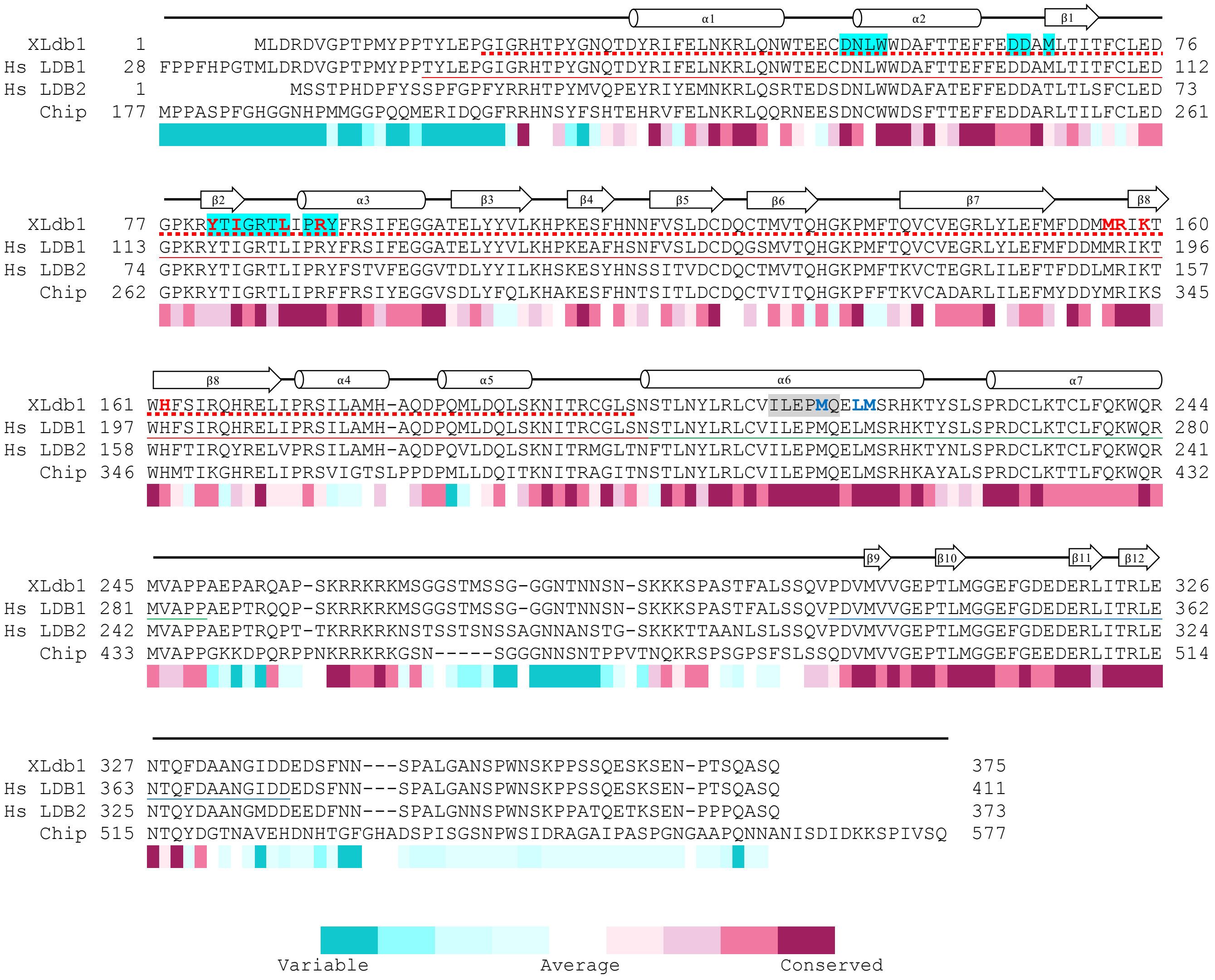

Fig. S4 Sequence comparisons of Chip/LDB orthologs

Sequence alignment of Chip/LDB orthologs, with conservation and structural elements shown (Hs, *Homo sapiens*); *cyan underlay*, DARPin-binding residues; *grey underlay*, sequence stretch essential for association between chicken Ldb1 and Ssdp1 or Ssdp2 [9]; *red*, Chip/LDB residues required for SSDP association; *blue*, LCCD α6 residues required for SSDP interaction; DD (*red line*), *Xenopus* DD used for crystallization (*red dashed line*), LCCD (*green*) and LID domain (*blue*) are underlined. Conservation score was calculated with ConSurf webserver [10].

Name	Clone	NXC	His$_6$ tag – PreScission protease site	N-Cap	1. Repeat
	Consensus		MRGSHHHHHHHHHHGGGGSYPYDVPDYALEVLFQGPGS	DLGKKLLEAAXXGQDDEVRILMANGADVNA	XDXXGXTPLHLAAXXGHLEIVEVLLKZGADVNA
DARPin1	1749_E2	N2C	....	..........RS..................	K.SF.S.......NV...........T......
DARPin2	1749_H7	N3C	....	..........QQ.HL...............	V.QV.H.......WQ...........T......
DARPin3	1749_B8	N2C	....	..........TE..................	H.RL.S.......KM...........T......
DARPin4	1750_H2	N2C	....	..........RA..................	A.NN.M.....P.EY...........A......
DARPin5	1750_H4	N3C	....	..........KM..................	M.QR.R.......WI...........T......
DARPin6	1750_E8	N2C	....	..........RA..................	S.QW.M.......VV...........T......
DARPin7	1750_C10	N2C	....	..........RA...N..............	K.WK.I.......QL...........T......
DARPin8	1750_D12	N1C	....	..........KH..................	T.YF.Y.......AE...........H......
DARPin9	1751_H1	N2C	....	..........YI..................	I.QQ.D.......MS...........T......
DARPin10	1752_G8	N3C	....	..........RA..................	H.KR.H.......TN...........T......
DARPin11	1753_E12	N3C	....	..........RA..................	F.WM.N.......WD...........T......
DARPin12	1754_A1	N2C	....	..........KQ.HL...............	Q.ER.E.......LW...........T......
DARPin13	1754_E5	N2C	....	..........MW..................	A.DI.V.......EE...........T......
DARPin14	1756_E11	N3C	....	..........MW..................	A.WI.T.......QY...........T......
DARPin15	1756_G11	N3C	....	..........RM..................	M.WV.Q.......FE...........T......

Name	Clone	NXC	2. Repeat	3. Repeat	C-Cap
	Consensus		XDXXGXTPLHLAAXXGHLEIVEVLLKZGADVNA	XDXXGXTPLHLAAXXGHLEIVEVLLKZGTDVNA	QDXXGXTPFDLAXXXGNEDIAEVLQKAAKLN
DARPin1	1749_E2	N2C	W.EF.Y.......FD...........H......		..MI.Q......AFA................
DARPin2	1749_H7	N3C	N.DT.I.......ME...........T......	K.WF.Y.......WA...........H.T....	..KF.K......IDN................
DARPin3	1749_B8	N2C	E.TA.Y.......AW...........H......		..KF.K......AIF................
DARPin4	1750_H2	N2C	Y.QF.Y.......YD...........H......		..SW.Q......AMR................
DARPin5	1750_H4	N3C	M.IL.E.......MD...........A......	M.RW.Y.......YD...........H......	..KF.K.A..ISIDN................
DARPin6	1750_E8	N2C	Y.MM.W.......W-...........H......		..KF.K......IDN................
DARPin7	1750_C10	N2C	H.DL.Y.......AE...........H......		..TW.N......AFM................
DARPin8	1750_D12	N1C			..RA.H......AAF................
DARPin9	1751_H1	N2C	Y.HW.Y.......NE...........H......		..KW.Q......AWH................
DARPin10	1752_G8	N3C	Y.RT.V.......MW...........A......	L.MV.Y.......WD...........H......	..KF.K......IDN................
DARPin11	1753_E12	N3C	A.FF.M.......ND...........H......	R.FS.H.......FT...........H......	..KF.K......IDN................
DARPin12	1754_A1	N2C	Y.VY.N.......TD...........H......		..RW.Q......AFF................
DARPin13	1754_E5	N2C	A.AL.W.......WA...........H......		..KF.K......AWS................
DARPin14	1756_E11	N3C	I.DF.I.......YQ...........A......	Q.QR.H.......TA...........H......	..KF.K......IDN................
DARPin15	1756_G11	N3C	A.TF.F.......HA...........T......	K.LS.H.......MQ...........H......	..KF.K......IDN................

Fig. S5 DARPin sequences

Sequence alignment of DARPins tested as crystallization chaperones. Randomised position in DARPins shown with grey underlay.

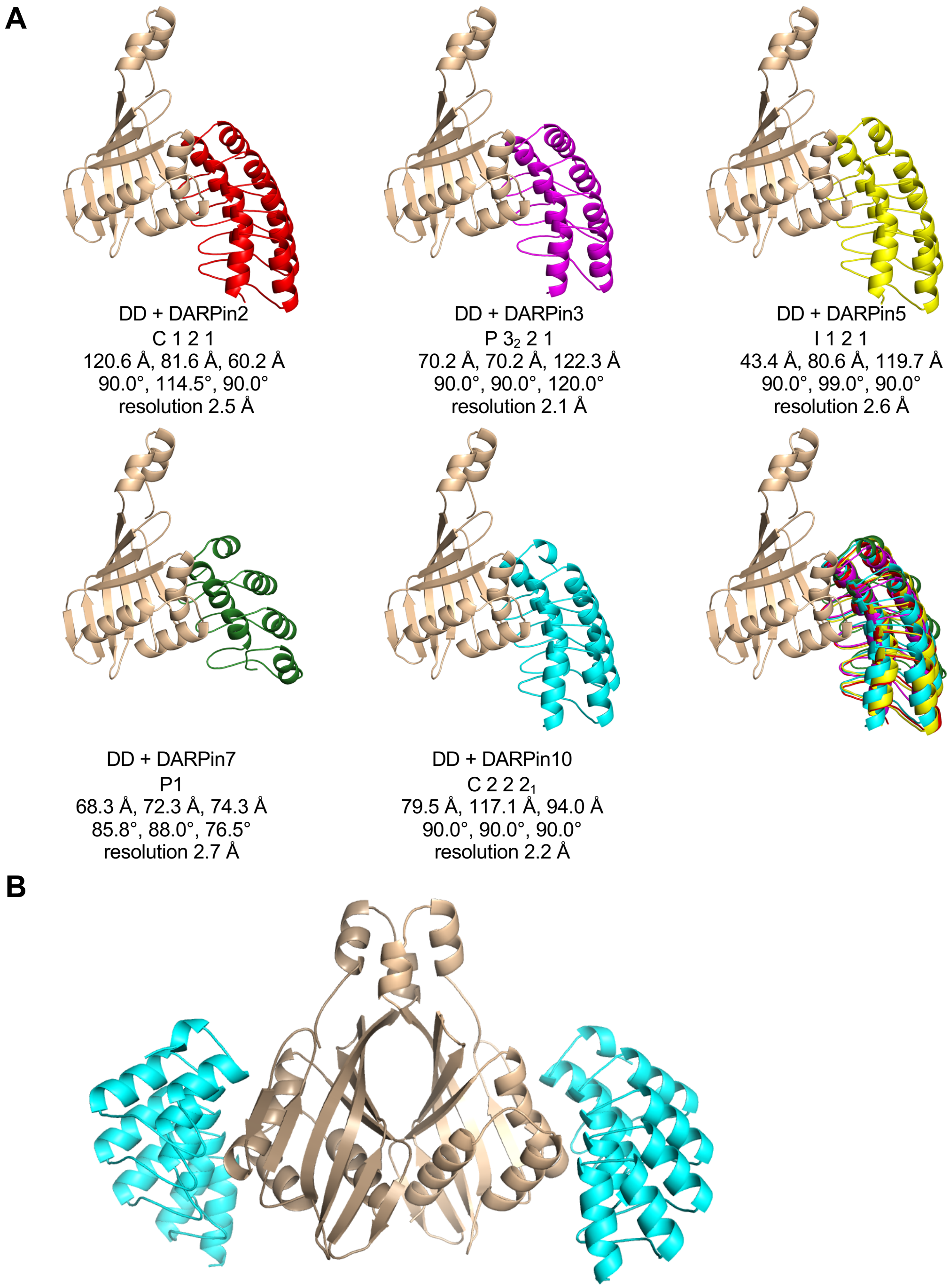

Fig. S6 Crystal structure of DD-DARPin complexes

(**A**) Crystal structures of all five DD-DARPin complexes determined, and superimposition with DD (*wheat*) of all structures. (**B**) Biological assembly of DD (*wheat*) in complex with DARPin10 (*cyan*); note that only the two highest-quality DD-DARPin structures (collected from optimized crystals) were submitted to PDB. The structures for the remaining three complexes (with DARPin2, 5 and 7) were lower-quality, but confirmed the recognition of the same epitope.

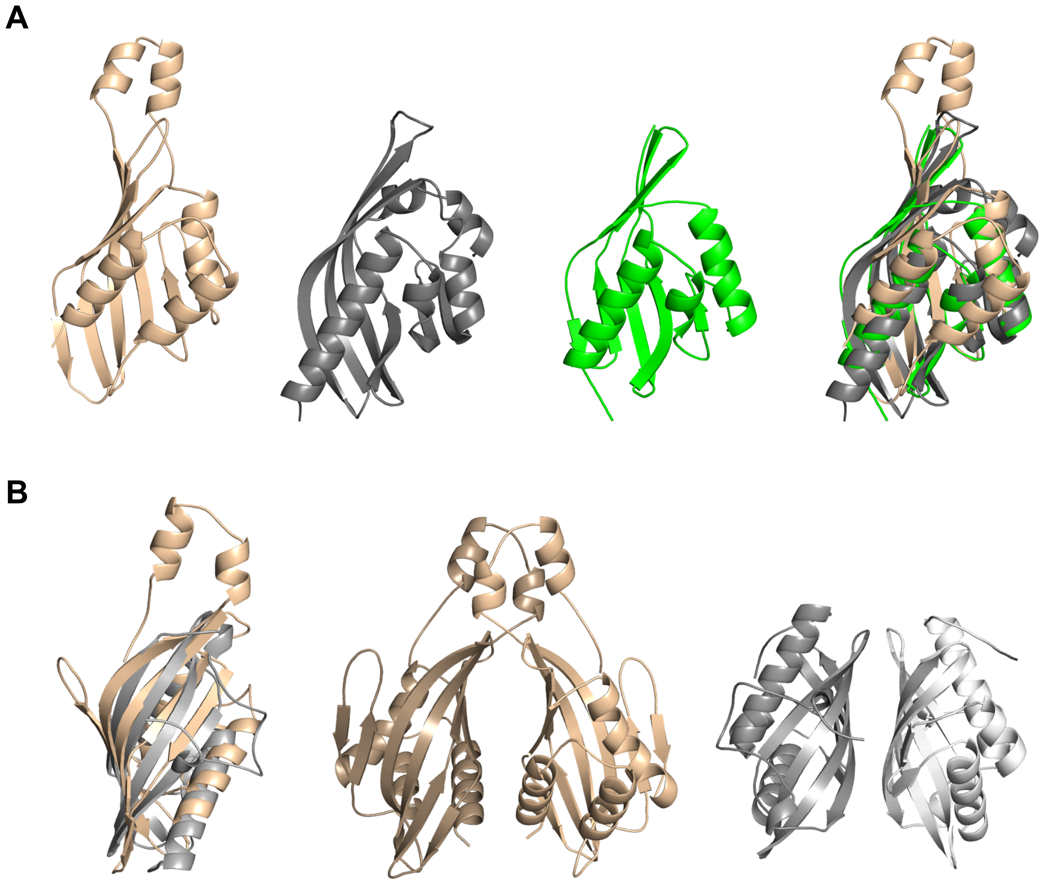

Fig. S7 Comparison between DD and bacterial ketosteroid isomerases
(**A**) Comparison of DD (*wheat*), scytalone dehydratase (1STD; *grey*) and ketosteroid isomerase (3NXJ; *green*); *right*, superimposition of all three proteins. (**B**) Different dimerization modes of DD (*wheat*) and protein PFL_3262 from *Pseudomonas fluorescens* (2IMJ; *grey*) which dimerizes via its curved β-sheets.

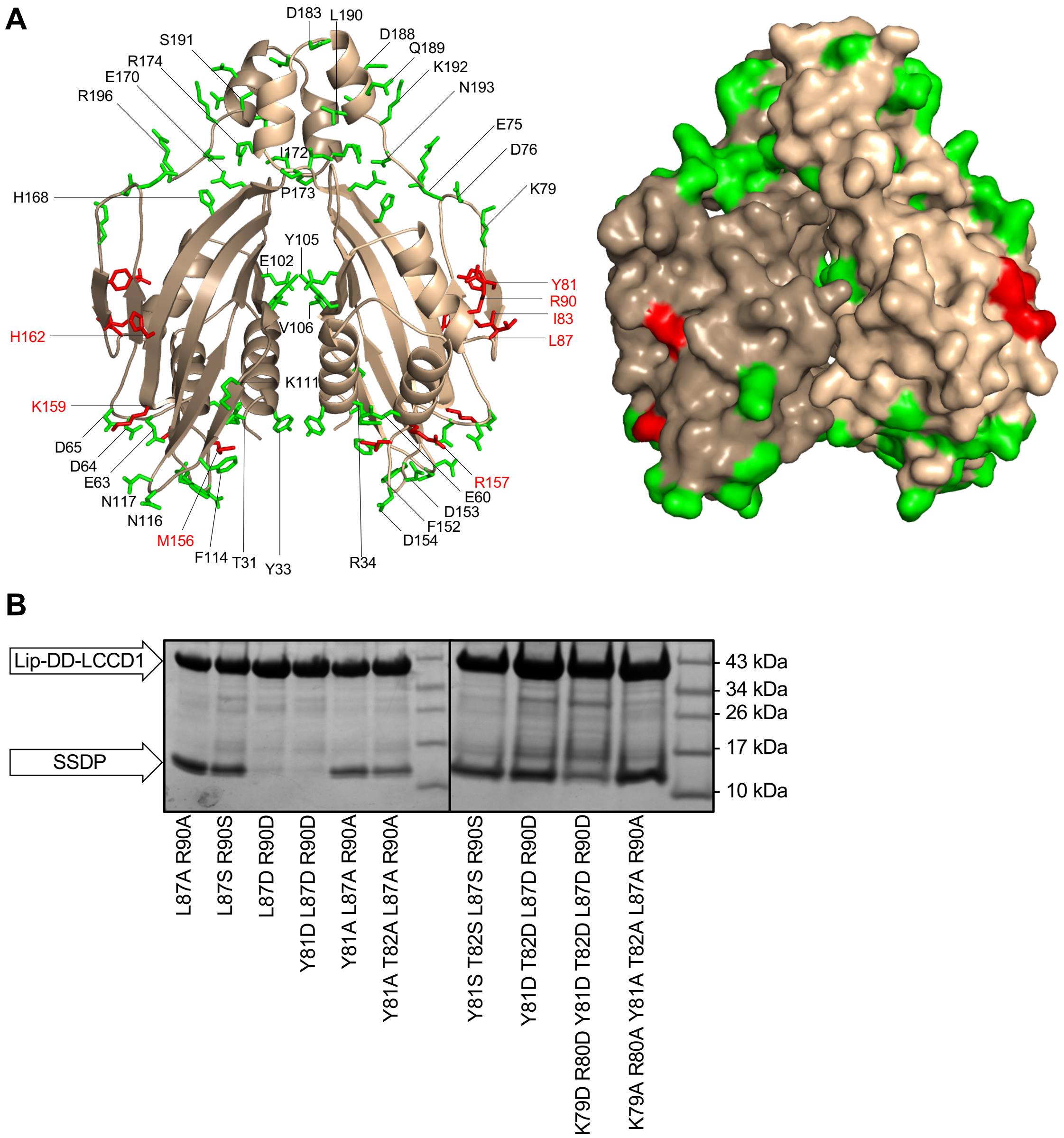

Fig. S8 DD residues tested for SSDP interaction

(**A**) Position of substitutions of solvent-exposed DD residues tested for interaction with SSDP (*red*, blocking or reducing interaction; *green*, no effect). (**B**) Pull-down assays between recombinant His$_6$-Lip-DD-LCCD1 and SSDP.

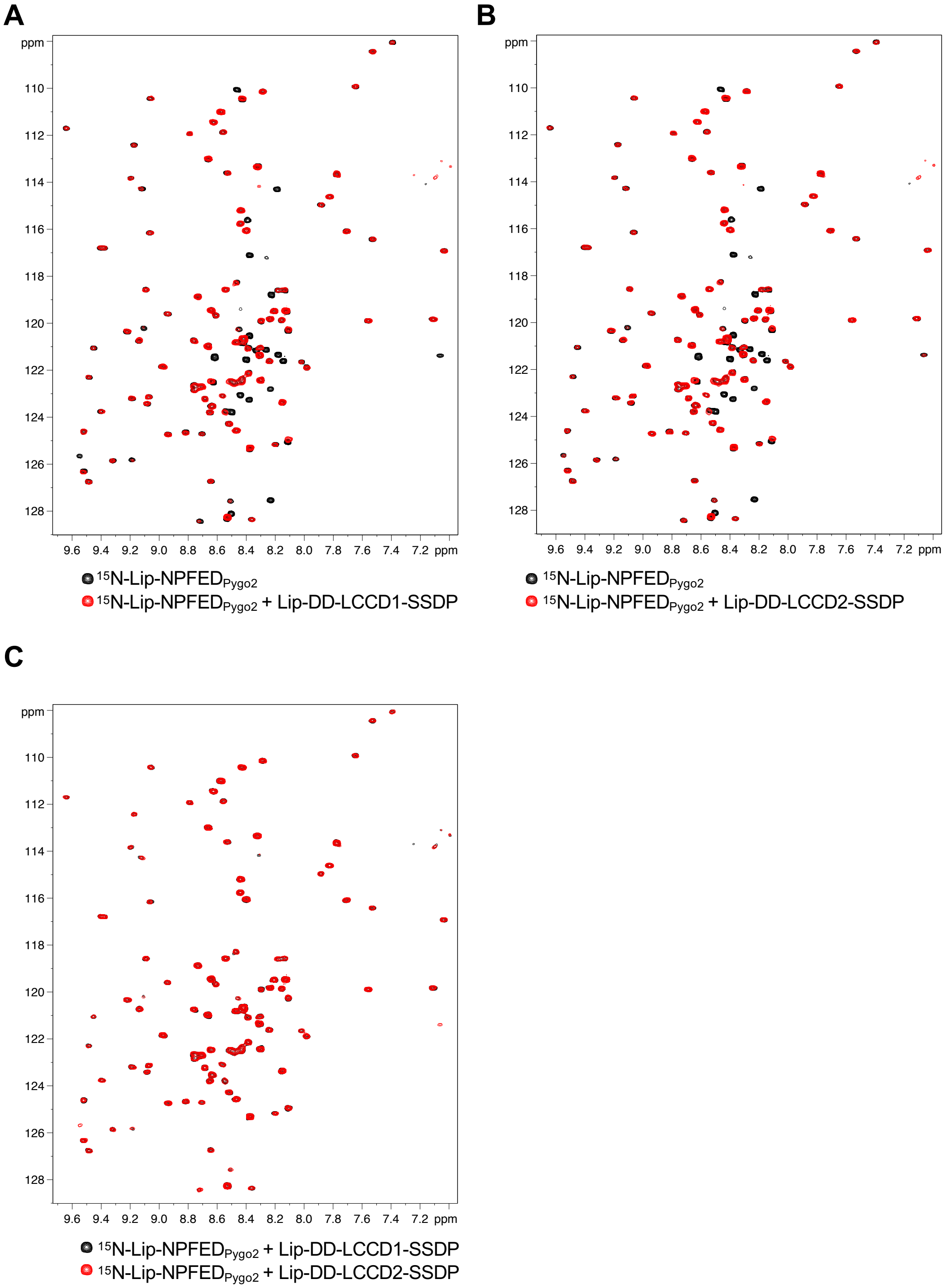

Fig. S9 Binding of Pygo2-NPFED to ChiLS

(**A, B**) Overlays of {^{1}H,^{15}N}-BEST-TROSY NMR spectra for 50 µM ^{15}N-labeled wt Lip-Pygo2$_{58\text{-}84}$ alone (*black*) or probed with (**A**) Lip-DD-LCCD1-SSDP or (**B**) Lip-DD-LCCD2-SSDP; see **Fig. S9*A***, for sequence of Pygo2$_{58\text{-}84}$. (**C**) BEST-TROSY overlay for 50 µM ^{15}N-labeled wt Lip-Pygo2$_{58\text{-}84}$ probed with Lip-DD-LCCD1-SSDP (*black*) or Lip-DD-LCCD2-SSDP (*red*).

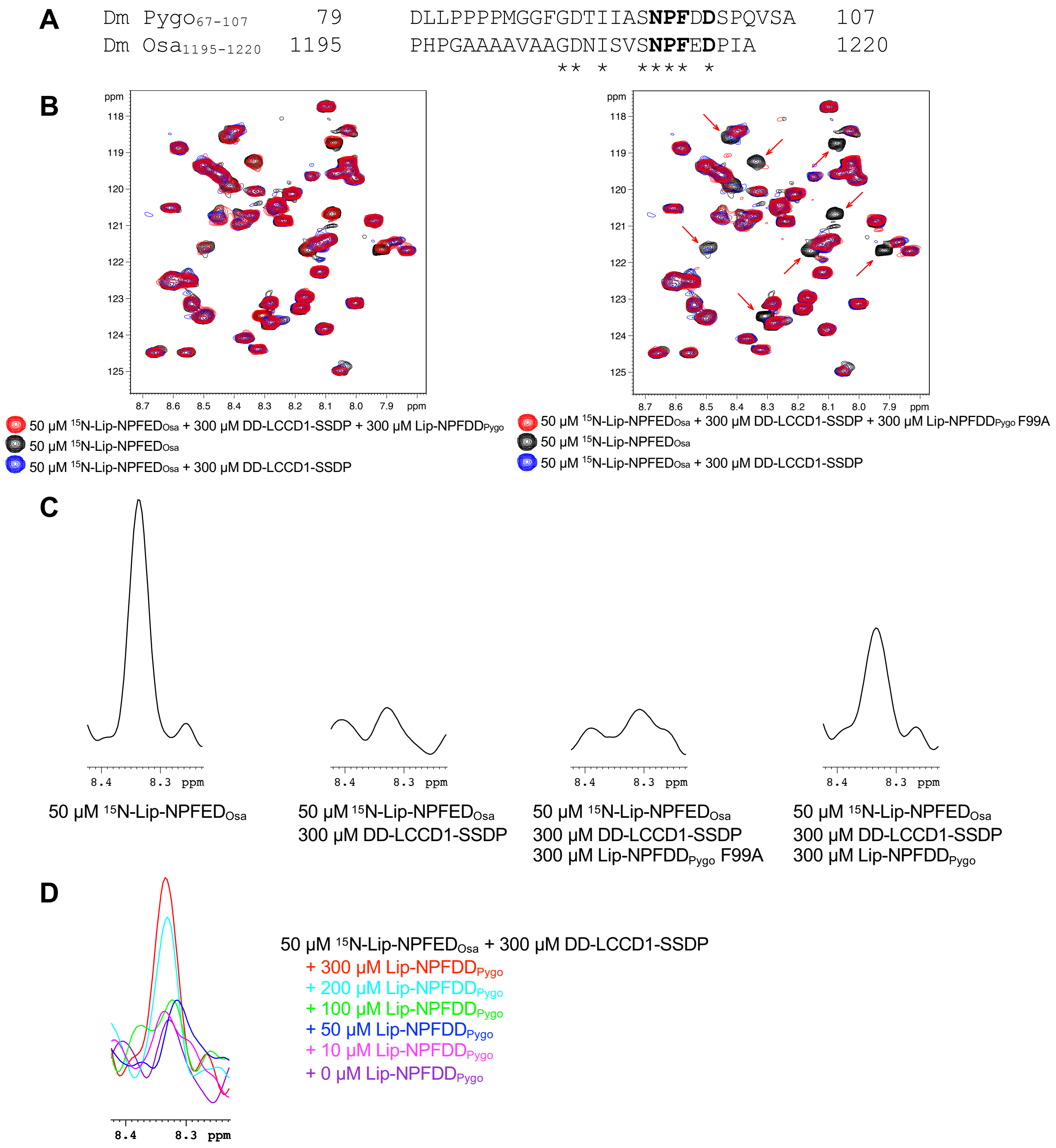

Fig. S10 Competition between Pygo- and Osa-NPFxD for binding to ChiLS

(A) Sequence alignment of NPFxD peptides of Pygo and Osa (Dm, *Drosophila melanogaster*). (**B**) Overlays of {^{1}H,^{15}N}-fHSQC NMR spectra for ^{15}N-labeled Lip-Osa$_{1195\text{-}1220}$ alone (*black*) and in the presence of 300 µM Lip-DD-LCCD1-SSDP (*blue*) and 300 µM wt (*left panel*) or F99A Pygo$_{67\text{-}107}$ (*right panel*). Red arrows indicate peaks from Osa$_{1195\text{-}1220}$ that are bleached by line broadening on addition of ChiLS. In each case, the line broadening is reversed as ChiLS is displaced by addition of wt but not F99A Pygo$_{67\text{-}107}$. (**C**) Peak height changes are illustrated using one-dimensional slices parallel to f_2 through the fHSQC datasets in (**B**) at 119.2 ppm ^{15}N frequency. (**D**) Overlay of slices as in (**C**), showing that wt Pygo$_{67\text{-}107}$ reverses ChiLS-induced line broadening of Osa$_{1195\text{-}1220}$ in a concentration-dependent manner.

	SSDP	DD – DARPin3	DD – DARPin10
**PDB ID**	6S9R	6S9T	6S9S
**Crystallization condition**	0.1 M MES,<br>pH 6.0-6.4<br>1.7-1.9 M $(NH_4)_2SO_4$<br>0.01 M $MgCl_2$	0.1 M TRIS<br>pH 8.5<br>20% PEG 8K<br>0.2 M Li_2SO_4	0.1 M TrisHCl<br>pH 7.0<br>32% PEG200<br>0.05 M Li_2SO_4
**Resolution range**	43.48 - 2.40<br>(2.486 - 2.40)	43.15 - 2.05<br>(2.123 - 2.05)	49.7 - 2.20<br>(2.279 - 2.20)
**Space group**	I 4 2 2	P 3_2 2 1	C 2 2 2_1
**Unit cell** (a, b, c / Å)<br>(α, β, γ / °)	137.50, 137.50, 53.86<br>90, 90, 90	70.22, 70.22, 122.26<br>90, 90, 120	79.51, 117.12, 93.97<br>90, 90, 90
**Total reflections**	264751 (25193)	890249 (87876)	297544 (28144)
**Unique reflections**	10414 (1010)	22528 (2198)	22606 (2223)
**Multiplicity**	25.4 (24.7)	39.5 (40.0)	13.1 (12.6)
**Completeness (%)**	99.5 (97.8)	99.92 (99.86)	99.14 (98.03)
**Mean I/sigma(I)**	33.4 (2.55)	23.53 (1.71)	15.6 (1.0)
**Wilson B-factor**	80.55	47.91	59.80
**R-merge**	0.053 (1.46)	0.127 (2.78)	0.094 (3.63)
**R-meas**	0.055 (1.49)	0.129 (2.81)	0.098 (3.78)
**R-pim**	0.011 (0.30)	0.021 (0.44)	0.027 (1.05)
**CC1/2**	1 (0.93)	1 (0.78)	0.99 (0.59)
**CC***	1 (0.98)	1 (0.94)	0.99 (0.73)
**Reflections in refinement**	10372	22514 (2195)	22443 (2187)
**Reflections used for R-free**	520	1066 (122)	1139 (103)
**R-work**	0.2248	0.218	0.233
**R-free**	0.2653	0.279	0.280
**Number of non-hydrogen atoms**	1004	2430	2610
**Macromolecules**	1004	2377	2604
**ligands**	0	26	0
**solvent**	0	27	6
**protein residues**	121	295	325
**RMS(bonds)**	0.015	0.015	0.015
**RMS(angles)**	1.91	2.03	2.00
**Ramachandran favored (%)**	94.02	96.56	95.95
**Ramachandran allowed (%)**	5.98	3.09	3.43
**Ramachandran outliers (%)**	0.00	0.34	0.62
**Rotamer outliers (%)**	6.12	1.58	3.24
**Clashscore**	6.61	5.88	3.31
**Average B-factor**	95.35	61.75	78.18
**macromolecules**	95.35	61.74	78.23
**ligands**	n/a	72.10	n/a
**solvent**	n/a	52.86	55.71
**Number of TLS groups**	12	10	14

Table S1. Data collection and refinement statistic for SSDP, DD-DARPin3 and DD-DARPin10. Statistics for the highest-resolution shell are shown in parentheses.

**Lip-DD-LCCD1**	**SSDP pulldown**
L87A R90A	++
L87S R90S	++
L87D R90D	-
Y81D L87D R90D	-
Y81A L87A R90A	+
Y81A T82A L87A R90A	+
Y81S T82S L87S R90S	++
Y81D T82D L87D R90D	++
K79D R80D Y81D T82D L87D R90D	+
K79A R80A Y81A T82A L87A R90A	++
K79S R80S Y81S T82S L87S R90S	++
ΔF72 ΔC73 ΔL74 ΔE75 ΔD76 P78G K79G R80G	++
ΔF72 ΔC73 ΔL74 ΔE75 ΔD76 P78G K79G R80G Y81A L87A R90A	++
ΔF72 ΔC73 ΔL74 ΔE75 ΔD76 P78G K79G R80G Y81D T82D L87D R90D	+

Table S2. XLdb1 mutants used for *in vitro* binding assays (++, strong binding; +, weak binding; -, residual binding)

XLdb1	Chip	SSDP binding
T31	T216E	++
Y33	H218E	++
R34	R219E	++
E60	E245R	++
E63 D64 D65	E248K D249K D250K	++
E75 D76 G77	E260K D261K G262T	++
K79	K264E	++
Y81	Y266D	+
Y81 L87 R90	Y266D L272D R275D	-
I83	I268D	-
L87 R90	L272D R275D	-
E102 Y104 V106	D287R Y289R Q291R	++
Y104 V106	Y289R Q291R	++
K111	K296E	++
F114	F299R	++
N116 N117	N301R T302R	++
Y152	Y337R	++
D153 D154	D338R D339R	++
M156 R157	M341R R342E	+
R157 K159 H162	R342D K344D H347D	-
H168 E170 R174	H353A E355R R359A	++
E170	E355K	++
E170 N193	E355C N379C	++
I172 R174	I357D R359D	++
P173 R174	P358D R359D	++
D183 P184	D369E P370E	++
D188 Q189	D374P Q375P	++
D188 Q189 L190 S191	D374I Q375R I376W T377E	++
K192	K378E	++
G198	G384R	++
R196 C197 G198 L199 S200 N201	R382L A383G G384E I385P T386P N387G	++
M216	M402E	-
M216 L219 M220	M402E L405D M406R	-
L219	L405D	+
M220	M406R	-
L244 L248	L420E L424E	+
R231 D232	R417E D418K	++
F239	F425E	++
K241	K427D	++
R231 D232	R417E	++

Table S3. Chip mutants used for cell-based coIP assays (++, strong binding; +, weak binding; -, residual binding). Corresponding residues in XLdb1 are shown on the left.

Supplementary References

1 Dreier, B. & Pluckthun, A. Ribosome display: a technology for selecting and evolving proteins from large libraries. *Methods Mol Biol* **687**, 283-306 (2011).

2 Pluckthun, A. Ribosome display: a perspective. *Methods Mol Biol* **805**, 3-28 (2012).

3 Zahnd, C., Sarkar, C. A. & Pluckthun, A. Computational analysis of off-rate selection experiments to optimize affinity maturation by directed evolution. *Protein Eng Des Sel* **23**, 175-184 (2010).

4 Sheldrick, G. M. A short history of SHELX. *Acta Crystallogr A* **64**, 112-122 (2008).

5 Langer, G. G., Hazledine, S., Wiegels, T., Carolan, C. & Lamzin, V. S. Visual automated macromolecular model building. *Acta Crystallogr D Biol Crystallogr* **69**, 635-641 (2013).

6 Emsley, P., Lohkamp, B., Scott, W. G. & Cowtan, K. Features and development of Coot. *Acta Crystallogr D Biol Crystallogr* **66**, 486-501 (2010).

7 Murshudov, G. N. *et al.* REFMAC5 for the refinement of macromolecular crystal structures. *Acta Crystallogr D Biol Crystallogr* **67**, 355-367 (2011).

8 Ran, F.A., Hsu, P.D., Wright, J., Agarwala, V., Scott, D.A. *&* Zhang, F. Genome engineering using the CRISPR-Cas9 system. *Nat Protoc*. **11**, 2281-2308 (2013)

9 Lee, B., Lee, S., Agulnick, A. D., Lee, J. W. & Lee, S. K. Single-stranded DNA binding proteins are required for LIM complexes to induce transcriptionally active chromatin and specify spinal neuronal identities. *Development* **143**, 1721-1731 (2016).

10 Ashkenazy, H., Abadi, S., Martz, E., Chay, O., Mayrose, I., Pupko, T. & Ben-Tal, N. ConSurf 2016: an improved methodology to estimate and visualize evolutionary conservation in macromolecules. *Nucleic Acids Res*. **44**, 344-350 (2016).
